# The Cortical Source of the P300 ERP Generator in the Associative Learning Task: A Consensus of Four Inversion Algorithms

**DOI:** 10.3390/brainsci16090953

**Published:** 2026-09-08

**Authors:** Daniyar Kalmagambetov, Mikhail Ye. Mel’nikov, Manzura Zholdassova, Altyngyl Kamzanova, Daniyar Abdilmanov, Gaukhar Datkhabayeva, James Eliassen, Jane B. Allendorfer, Almira Kustubayeva

**Affiliations:** 1 Department of Biophysics, Biomedicine, and Neuroscience, Farabi University, Almaty 050040, Kazakhstan; 2 Brain Institute, Farabi University, Almaty 050040, Kazakhstan; 3 Bosch Automotive Steering, Florence, KY 41042, USA; 4 Department of Neurology, University of Alabama, Birmingham, AL 35294, USA

**Keywords:** associative learning, event-related potentials (ERP), development, source localization, middle temporal gyrus, angular gyrus

## Abstract

**Highlights:**

**What are the main findings?**

- Four inversion algorithms for Early > Late-stage associative learning localized a cue stimulus onset-locked P300 in the right inferior parietal lobule and angular gyrus.
- The four-algorithm conjunction of a feedback-related P300 for Early > Late-stage associative learning is localized in the right occipital cortex and bilateral temporal regions, including middle temporal gyrus and superior temporal gyrus/Heschl’s gyrus.
- No cortical source-level effect of participant age was significant under the multi-algorithm consensus criterion.

**What are the implications of the main findings?**

- Temporal-lobe involvement, alongside occipital components, are more engaged in context and feedback processing in the early stage of the associative learning process, before rule application becomes automated.

**Abstract:**

**Background:** Associative learning, the process of binding stimuli, responses, and outcomes, is critical for behavioral adaptation. While event-related potentials (ERPs) like the P300 effectively index the cognitive effort and context updating required during trial-and-error learning, the precise cortical generators of these signals and their shift across development remain difficult to isolate due to the electroencephalography (EEG) inverse problem. The aim of the study was to examine the differences in P300 localization depending on the associative learning task stage and participants’ age. **Methods:** A total of 148 participants (aged 7–21) completed a visual-motor associative learning task while undergoing 64-channel EEG recording. Source localization was performed over the 300–500 ms (P300) time window as a conjunction of results of four inversion algorithms at FWE-corrected *p* < 0.05: multiple sparse priors with greedy search (GS), independent and identically distributed (IID), low-resolution electromagnetic tomography (LOR), and empirical Bayes beamformer (EBB). Exploratory three-algorithm (IID, LOR, and EBB) conjunction analysis results were also reported. **Results:** A spatial convergence analysis of Early > Late-stage differential maps revealed a consensus across all four models for both cue Onset-locked (right inferior parietal lobule/angular gyrus) and Feedback-locked (occipital, temporal (including bilateral middle temporal gyrus), and orbitofrontal regions) activity. The exploratory three-algorithm analysis additionally implicated left middle temporal gyrus and angular gyrus for cue Onset-locked rule acquisition. No results were produced for age-related effect surviving either the four-algorithm or three-algorithm consensus criterion. **Conclusions:** This multi-algorithm consensus links active rule search to distributed cortical pattern, involving angular (for cue stimulus processing) and middle temporal gyri (for feedback processing), while no age-related changes in these areas have been demonstrated.

## 1. Introduction

Associative learning, the process of binding information on stimuli, responses, and outcomes into predictive representations, plays a fundamental role in behavioral adaptation [1,2]. It comprises two stages: an early stage (rule acquisition) involving initial trial-and-error to deduce rules; and a late stage (rehearsal) entailing application of learned associations [1].

The associative learning paradigm typically includes a sequence of discrete events such as stimulus demonstration, subject reaction, and feedback reception [1,2]. Event-related potentials (ERPs), neural electrical activity that can be measured with electroencephalography (EEG), provide valuable information regarding the temporal dynamics of cognitive processes in associative learning with high temporal resolution [3]. ERP’s excellent temporal resolution make it well suited for precise characterization of the neural underpinnings of certain stages and routines within cognitive acts, and EEG’s portability, accessibility, and absence of strong contraindications (relative to functional magnetic resonance imaging, fMRI) enable large-scale ERP phenotypic characterization pursued under neurocognitive phenomics frameworks, an approach still comparatively underexploited, compared to fMRI-based research [4]. This scalability is particularly relevant to developmental cognitive neuroscience, where EEG’s tolerance of movement and applicability across a wide age range offer practical advantages over fMRI [5].

Among ERP components, the P300 (or P3) wave is typically elicited during decision-making and is particularly informative as it is strongly modulated by stimulus and feedback significance, as well as by expectancy [6,7]. The P300 is also associated with stimulus evaluation, context updating, attention allocation, and working-memory updating [8,9]. Given that the P300 indexes engaged processing capacity under task demands, it is often used as a proxy for cognitive effort and load in learning and decision-making research [10]. In learning paradigms, P300’s properties change as contingencies are acquired, consistent with shifting from a reliance on external feedback to using the obtained rules once mappings stabilize [6,7].

Importantly, P300’s characteristics appear to change across development [11,12]. Developmental ERP research in feedback processing and learning shows systematic age-related differences in the P300’s amplitude and latency consistent with maturation-related changes in the allocation of processing resources during learning and the evaluation of outcomes [12,13,14]. Because P300’s amplitude has been argued to scale with engaged processing capacity and resources under task demands [10], these developmental effects are commonly interpreted in terms of age-related shifts in cognitive effort and efficiency.

To better understand the neural origins of the ERP components including the P300, source localization techniques are employed. To connect the surface ERPs to specific neural generators, intracranial current distributions are computed that best explain the observed sensor-level potentials under biophysical forward models [15,16]. In practice, this is operationalized via families of inverse solutions with different priors and constraints like distributed minimum-norm approaches (e.g., dSPM) [17,18], smoothness-constrained tomographic solutions such as Low-Resolution Electromagnetic Tomography (LORETA) [19], sparse Bayesian formulations such as Multiple Sparse Priors (MSP) [20], and spatial filtering/beamforming [21,22]. By applying source localization to EEG data obtained during associative learning tasks, one can identify the brain regions involved in generating specific ERP components such as the P300. This allows for a more detailed understanding of the neural mechanisms underlying cognitive processes [15,16]. Critically, recent evidence demonstrates that individual differences in associative learning performance covary with distinct ERP topographies, allowing direct mapping of the underlying cortical generators that drive learning capacity using EEG source localization [23].

The existing EEG/ERP localization data suggest the involvement of several cortical areas in associative learning, including the prefrontal/orbitofrontal cortices [23,24,25], anterior cingulate cortex (ACC) [24], different parietal regions [24,25], and middle temporal gyrus (MTG) [24]. Notably, although the P300-family is widely used to assess outcome evaluation and learning-related updating, generator differences specific to learning stages (e.g., acquisition or rehearsal) and how generators change with age are not yet well characterized in the associative learning ERP literature [3].

The converging fMRI evidence implicates a distributed network involving corticostriatal and frontoparietal circuits in feedback-guided learning and associative rule formation. Across related reinforcement and/or associative learning tasks, learning- and feedback-sensitive activity has been reported in the striatum [2,26,27,28], along with lateral prefrontal cortex and medial frontal cortex regions including the ACC and pre-supplementary motor association cortex that are typically engaged when rules are being inferred, updated, and implemented [2,11,29]. Additional contributions have been reported from the anterior insula and hippocampus [27,30] and temporal regions, including the MTG and angular gyrus (AG) (e.g., [31,32,33,34]). The fMRI and EEG findings partially match, as both modalities support the involvement of overlapping prefrontal areas and MTG in feedback-guided associative learning, while fMRI further delineates contributions from striatal, insular, and temporal regions.

Despite ongoing progress in the field, EEG source reconstruction remains limited by the classical inverse problem, sensitivity to head model assumptions, and dependence on priors and constraints which compromise precision or even the validity of solutions [15,35,36]. These challenges motivate the use of multi-algorithm strategies (cross-validating across inverse solutions) when anatomical inferences are central to the research question. To address these source reconstruction issues, the current study employed a multimodal approach that leverages the complementary strengths of different source reconstruction methods. The combined use of distributed source models such as minimum norm estimates (MNE), smooth spatial priors (LORETA-like, LOR), MSP, and beamformers, as well as the application of individual MRI-informed forward modeling might enhance the statistical robustness and spatial accuracy of P300 localization for a paradigm. Employing this approach, the current study aims at testing two main hypotheses: (1) associative learning regions like MTG and AG show greater P300 potential amplitude localization on the early stage compared to the late one; and (2) the amplitude values of the between-stage differences P300 signals in the MTG and AG are negatively correlated with age.

## 2. Methods

### 2.1. Participants

**Figure 1:**
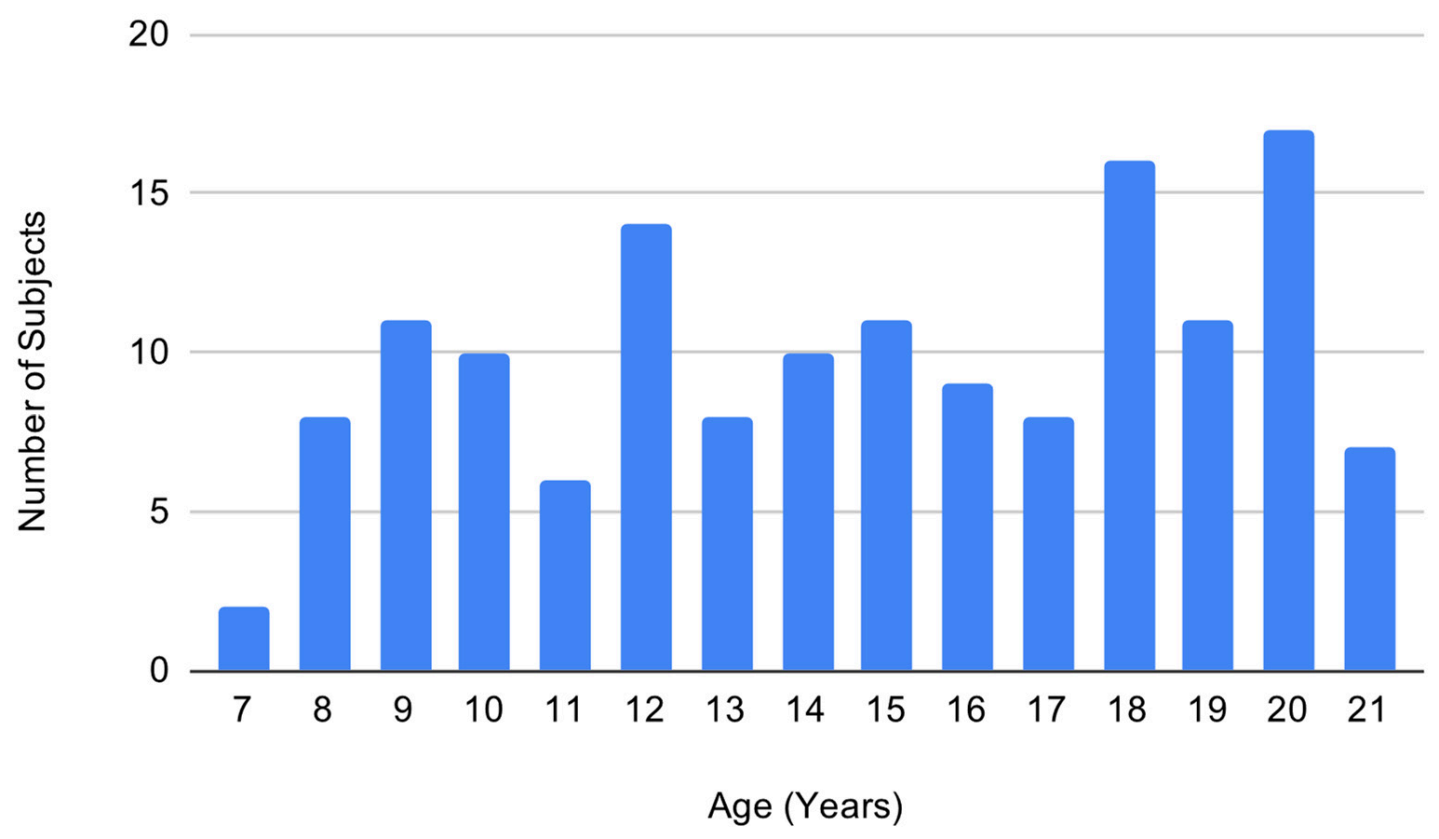
The distribution of the participants’ age.

Participants were recruited via public advertisements and word-of-mouth. To be eligible for the study, individuals had to meet the following inclusion criteria: aged between 7 and 21 years, be free from any history of diagnosed neurological or psychiatric disorders, be currently unmedicated and not undergoing active medical treatment, have no research MRI contraindications including pregnancy, cardiac pacemakers, or ferromagnetic implants, and demonstrate a willingness to comply with instructions and participate in all stages of the study. Participants were excluded from the analysis if they failed to follow the personnel instructions or voluntarily withdrew from the study at any stage. The data of 149 participants were analyzed; one subject was excluded from the final analysis due to a singular Boundary Element Method (BEM) matrix during forward solution computation, which prevented the accurate generation of the lead field matrix. The final cohort consisted of 148 participants (75 males, 73 females) with a mean age of 14.7 years (SD = 4.06) (Figure 1). Based on their primary language of instruction at school, 69 participants were identified as Kazakh-speaking and 79 as Russian-speaking. This study was conducted in full accordance with the Declaration of Helsinki. The research protocol received approval from the Local Ethics Committee of the al-Farabi Kazakh National University. Prior to data collection, all participants provided written informed consent. For participants under the age of 18, written informed consent was obtained from a parent or legal guardian.

### 2.2. Experimental Task Design and Procedure

The associative learning task followed a structured trial-and-error paradigm designed to experimentally dissect the rule acquisition and rehearsal stages. Each trial began with the display of a central fixation cross for 1.03 s, followed by a stimulus prompt presented on either the left or right side of the monitor for 2.25 s. Participants were instructed to respond within this 2.25 s window via left or right arrow keys based on a covert, deterministic logical rule. For each run, the active rule was drawn from one of four possibilities: “always left,” “always right,” “at prompt side” (spatially congruent with the cue), or “contrary to prompt side” (spatially incongruent). Following a 0.53 s post-response delay, visual feedback was provided for 2.0 s indicating rule conformity (correct/incorrect), though no monetary rewards were offered. The reward sensitivity and incentive valuation undergo non-linear maturation across the 7–21 age span [37,38]; therefore, not including monetary reward prevents the addition of a complex confounding effect to our data. Non-monetary reward types have also been shown to engage neural reward circuitry that overlaps substantially with that engaged by monetary reward [39], supporting the use of correctness feedback alone in the present paradigm.

A run consisted of 5, 6, or 7 consecutive trials sharing the same rule, with pseudorandomized run lengths preventing participants from predicting the end of a run. Changes in the cue stimulus signaled the start of a new run and prompted the search for a new rule. The experiment was organized into four blocks, each containing 8 runs (32 runs total), resulting in a total of 192 trials over approximately 18.6 min. Prior to the recording session, participants completed practice trials to ensure comprehension, and the paradigm was implemented using E-Prime v2.0.10.182 software (Psychology Software Tools Inc., Pittsburgh, PA, USA).

Behavioral performance was logged via E-Prime, allowing runs and trials to be classified based on accuracy and learning stage. Correct runs were defined by a 100% accuracy rate from the fourth trial onward, signaling successful rule acquisition; runs failing to reach this criterion were excluded from stage-specific ERP analyses. Within the correct runs, trials were categorized by stage: the Early stage (trials 1–3) represented active rule search and initial acquisition, while the Late stage (trials 4 and beyond) represented rule rehearsal, stabilization, and consistent application within correct runs.

### 2.3. Data Acquisition

EEG data were acquired in a sound-attenuated, electrically shielded facility at Kazakh National University, where participants were seated at a comfortable distance from a 20-inch, 60 Hz LCD monitor (LG Flatron 1942S; LG Electronics, Seoul, Republic of Korea). Signals were recorded using an eego mylab 64-channel amplifier (ANT Neuro, Hengelo, The Netherlands) and 10-10 system WaveGuard 64-channel Ag/AgCl electrode cap. During recording, CPz served as the online reference and AFz served as the ground, with electrode impedances maintained below 20 kΩ. Data were sampled at 4096 Hz via ANT Neuro eego v1.9.1 software (eemagine Medical Imaging Solutions GmbH, Berlin, Germany).

To facilitate source localization and anatomical alignment, structural T1-weighted images were acquired using a 3T GE SIGNA Architect scanner (GE Healthcare, Chicago, IL, USA) with a 3D BRAVO sequence. The acquisition parameters included a repetition time (TR)/echo time (TE) = 7.716 ms/3.14 ms, and a flip angle of 8°. The resulting images featured a voxel size of 0.4688 × 0.4688 × 1.2 mm, with a slice thickness of 1.2 mm and a slice intersection of 0.6 mm, totaling 260 sagittal slices in a 512 × 512 matrix.

### 2.4. EEG Preprocessing

**Figure 2:**
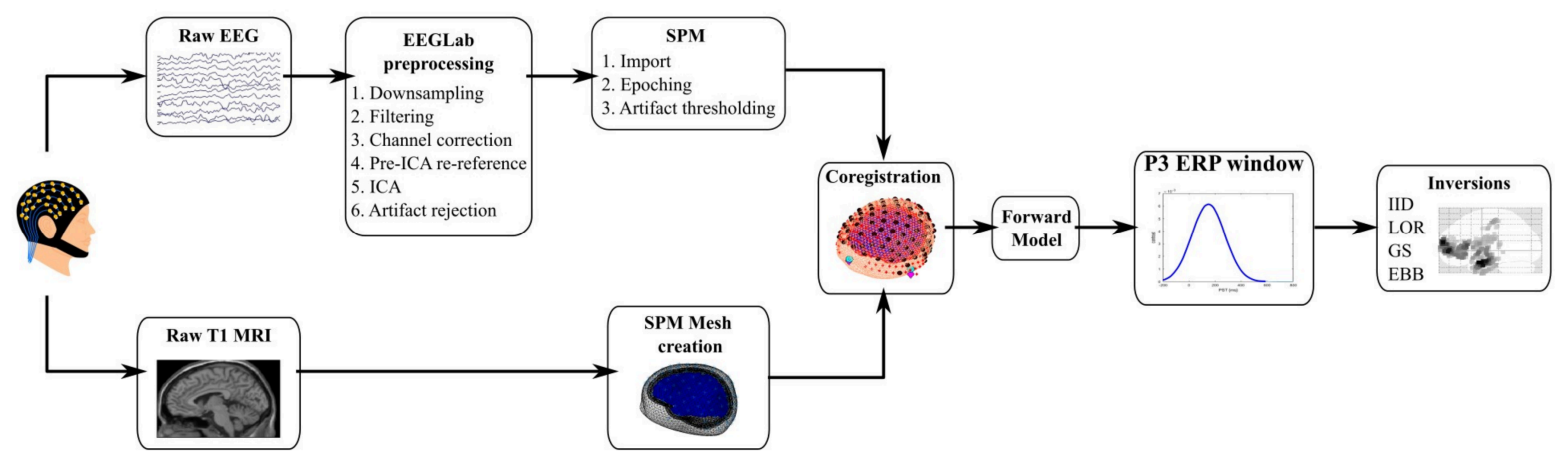
Multimodal data processing and source reconstruction pipeline. Schematic representation of the analytical workflow applied to the dataset. The pipeline details the sequential stages of data preprocessing, anatomical coregistration, and the implementation of source inversion algorithms for spatial localization of neural activity.

Offline preprocessing was conducted using a combination of MATLAB 2021b, the EEGLab 2024.0 toolbox, and SPM12 r7771 (Wellcome Centre for Human Neuroimaging, University College London, London, UK) (Figure 2). The pipeline involved downsampling the raw data to 512 Hz and applying a 0.3–40 Hz bandpass finite impulse response (FIR) filter using default EEGLab settings. The corrupted channels were identified via the CleanRawData function based on a correlation threshold of less than 0.7 with neighboring channels; these channels were then spatially interpolated using spherical splines. Next, the data were re-referenced to the average reference, and the Fp1, Fp2, and Fpz electrodes were excluded to minimize ocular artifact contamination during independent component analysis (ICA). To further optimize the ICA decomposition, the dataset was high-pass filtered at 1 Hz before applying the Infomax (runica) algorithm. The resulting ICA weights were then applied to the original 0.3 Hz high-pass-filtered data [40]. ICA components were subsequently classified using ICLabel 1.6, where any components with a chance of 88% or higher of belonging to eye, muscle, or channel noise categories were marked for rejection. In cases of excessively contaminated files, additional components were marked manually. In the final processing stage, original event markers were replaced with ones from the E-Prime logs to define four condition-specific bins: stimulus onset for early-stage correct runs, stimulus onset for late-stage correct runs, feedback onset for early-stage correct runs, and feedback onset for late-stage correct runs. For brevity, these are referred to throughout the remainder of the manuscript as “Onset” (i.e., stimulus onset, as distinct from feedback onset) and “Feedback” (i.e., feedback onset).

### 2.5. Source Reconstruction and Inversion Algorithms

The preprocessed EEG data were imported into SPM12 for source localization, where they were referenced to the average and epoched from −200 to 800 ms relative to stimulus or feedback onset. Any epochs containing amplitudes exceeding ±130 μV were discarded from the analysis in a corresponding channel. The number of good trials per subject per event type was extracted for subsequent usage as a covariate. Sensor-level signal-to-noise-ratio (SNR) was quantified over the 300–500 ms analysis window as shown in Equation (1) below, where root-mean-square (RMS) signal represents the baseline-corrected RMS amplitude of the robust grand-average waveform within the time window of interest, and standard error (SE) noise (Equation (2)) denotes the SE derived from the weighted across-trial residual standard deviation pooled across the time window. The SNR averaged across channels was employed as a covariate in a subsequent analysis. For each participant, a fine-scale cortical mesh was generated from their individual T1-weighted structural MRI data, and electrode positions were co-registered to each subject’s cortical mesh using a template montage rigidly aligned via the nasion and left/right preauricular fiducial points. A three-shell (scalp–skull–brain) boundary element head model was constructed for each subject using SPM12’s default procedure.

(1) $$\mathrm{SNR}=\frac{{\mathrm{RMS}}_{\mathrm{signal}}}{{\mathrm{SE}}_{\mathrm{noise}}}$$

(2) $${\mathrm{SE}}_{\mathrm{noise}}=\frac{{\mathrm{SD}}_{\mathrm{noise}}}{\sqrt{n_{\mathrm{good}}}}$$

The conductivities for the brain and scalp were set at 0.33 S/m. Given the skull conductivity impact on the source localization outcomes [41] and its prominent decrease with age [42], in the current study it was calculated from participant’s age based on a linear regression formula [σ (skull conductivity, S/m) = 0.00977 − age in years × 0.00013 S/m], derived from the data [42]. To assess the sensitivity of source localization to assumed skull conductivity, the forward model was also recomputed using six fixed values from 0.003 to 0.008 S/m in steps of 0.001 S/m. All inversion algorithms and second-level analyses were run identically across the assumed skull conductivity values. To provide the estimates of results sensitivity to conductivity value on the cluster level (added to the entire contrast-level estimates), for each fixed conductivity, the cluster corresponding to the individualized model’s largest cluster was identified by voxel overlap where present, or otherwise as the nearest cluster peak. The peak displacement was additionally expressed relative to the four-way minimum-statistic map’s (see Section 2.6 below) own spatial smoothness (~23 mm full width at half-magnitude, FWHM for Onset, ~24 mm FWHM for Feedback) as a resolution-referenced measure.

The construction of the four-element BEM failed because of the small distance between the cortical surface and the skull internal surface. To explicitly address cerebrospinal fluid (CSF) conductivity, we tested a 5-compartment finite element model (FEM) with conductivity values set of 0.33, 1.79, 0.4, and 0.2 S/m for scalp, CSF, grey matter (GM), and white matter (WM), respectively, and the same skull conductivity range as the 3-element BEM model. However, the sharp change in conductivity ratio on the CSF–skull border invalidated the FEM models, resulting in <2% of the data variance explained in any combination of inversion type and skull conductivity.

After the leadfields computation, a group inversion was performed to align the functional data across all participants into a common source space, thereby optimizing the subsequent second-level analysis. Group inversion was performed in four chunks of 37 subjects each, with an identical chunk partition used for every inversion algorithm. Four distinct inverse modeling algorithms were applied over a 300–500 ms time window. The source amplitude was quantified as source power, computed after spatial smoothing (Gaussian kernel of 8 mm FWHM). All four algorithms used SPM12’s default dipole orientation handling for the canonical cortical mesh.

The inversions were computed separately for the four experimental conditions involving early and late-stage stimulus onsets and feedback presentations. The first model, MNE implemented in SPM12 as the Independent and Identically Distributed (IID) [17], identifies a source distribution that minimizes the total energy (L2 norm) required to explain the sensor-level data by treating all potential source locations as independent and equally probable. The second model, LOR, incorporates a constraint of spatial smoothness by applying a discrete Laplacian operator to the source space, which ensures neighboring voxels are highly correlated and provides depth compensation [19]. The third model utilized the multiple sparse priors (MSP) with Greedy Search (GS) approach, which employs a hierarchical Bayesian framework and a dictionary of potential source patches to adaptively transition between focal and distributed representations via restricted maximum likelihood [20]. Finally, the Empirical Bayes Beamformer (EBB) was applied to integrate spatial filtering with Bayesian inference, deriving the source covariance prior empirically from the sensor-level data to maintain high localization accuracy [22].

By employing a multi-model approach to source reconstruction, this study sought to minimize the bias inherent in any single mathematical inverse solution. The convergence of results across IID, LOR, GS, and EBB models ensures that the identified cortical regions of interest (ROIs) are robust to different physiological and mathematical assumptions. This comprehensive pipeline spanning from high-density EEG acquisition and individual MRI-informed forward modeling to sophisticated Bayesian inversion provides a rigorous framework for mapping the spatiotemporal dynamics of associative learning.

Because beamformers are known to underperform when sources are correlated [43,44] and effort is put in building beamformers robust to sources correlation [44,45], we assessed the suitability of EBB for this dataset in two ways (detailed in the Results): first, by comparing the resolution matrix (point-spread function per source) of all four main-analysis algorithms against six reference estimators (MNE, depth-weighted MNE, dSPM, sLORETA [46], eLORETA [47], and unit-noise-gain Linearly constrained minimum variance, LCMV) in SPM’s reduced spatial-mode space; second, by comparing model evidence (variational free energy) between EBB and SPM’s correlated-source beamformer variant, EBBcorr [45], which is designed to address this limitation directly. Related recent work has extended multi-source spatial-filtering frameworks of this kind to the MNE-Python ecosystem [44].

To assess the sensitivity of source localization to possible electrode shifts inevitable in case of fiducial-based alignment, electrode positions were systematically perturbed and the pipeline re-run to quantify the resulting change in source estimates. Perturbations were applied tangentially along the scalp surface (rotation about the head center, re-projected onto the scalp) to avoid the cancellation of perturbation effects by the forward model’s projection of electrodes onto the scalp. The perturbation magnitude was defined as the post-projection angular displacement scaled by local scalp radius. An initial subject-level screen was performed across a range (0.1–24.3 mm) of perturbation magnitudes to characterize the sensitivity of the forward model. A subset of representative perturbation magnitudes was then propagated through the full group-level inversion and second-level statistical pipeline. Specifically, we tested LOR as a distributed localization example across shift magnitudes and additionally GS as a deviant, more local solution algorithm at the largest magnitude, with model assuming individual skull conductivity. Therefore, we assessed the resulting impact of electrodes’ mispositioning on group-level statistical maps. One participant was excluded from this analysis because of a boundary-element geometry issue, leaving 147 subjects. The nine group-level arms (electrode shift magnitudes) comprised: a near-zero floor (0.1 mm, pitch axis); four fixed pitch-axis magnitudes (10, 13, 16, and 20 mm—cap riding forward/back about the left-right axis); a realistic per-subject arm, using a randomized displacement magnitude and axis drawn independently for each subject; and two age-graded arms, in which mean electrode displacement was linearly interpolated across the sample’s observed age range up to 15 mm in opposite directions (larger displacement in older versus younger participants, respectively), to assess the coregistration error scaling with age. To additionally assess the results robustness, re-smoothing the exported amplitude source images to four further kernel widths spanning 8.25–16 mm effective FWHM (from the initial 8 mm export kernel) for individual skull conductivity model and re-running the analysis at each level was performed.

### 2.6. Statistical Analysis

Group-level statistical analysis was implemented in SPM12 using a general linear model (GLM) framework to identify significant cortical activity associated with rule acquisition and maturation. For each combination of the four inversion methods (IID, LOR, GS, and EBB) and two event types (stimulus onset and feedback presentation), a separate second-level GLM was built. Given that SPM’s GLM cannot accommodate paired designs with continuous subject-level covariates, we first prepared individual Early vs. Late-stage contrasts (differential images) and then include them in a group analysis. This model featured correct number of degrees of freedom and handled the continuous covariates. Age was entered as the covariate of interest, while Language, Sex, Good trial count (summed for Early and Late stages), and average SNR were entered as covariates of no interest. In addition to main analysis, based on original localization images and assessing source power amplitude differences, the second analysis was run, based on scaled images and reflecting relative topographic distribution. All results were thresholded at a voxel-level *p* < 0.05, corrected for multiple comparisons using Family-Wise Error (FWE).

Agreement across inversion algorithms was formally assessed using minimum-statistic conjunction inference [48] over a combined explicit analysis mask of 142,799 voxels, with the common threshold defined by the most conservative whole-brain voxel-level FWE-corrected threshold (*p* < 0.05) across the algorithms in the conjunction. Conjunction across all four inversion algorithms (GS, LOR, IID, EBB) was evaluated as the primary consensus analysis. Because the Multiple Sparse Priors with Greedy Search (GS) algorithm enforces a focal/sparse prior with a restricted search volume (~61% of the mask; 86,962 voxels), the four-way conjunction is heavily constrained by this search boundary; conjunction across the three distributed/dense-prior estimators alone (LOR, IID, EBB), which is not bounded by GS’s search volume, is reported in parallel as an exploratory, more permissive companion analysis. We also quantified pairwise spatial agreement between all four algorithms using the Dice coefficient, with bootstrap 95% confidence intervals (1000 resamples) and a spatial-permutation *p*-value (1000 permutations, circular map shift).

To further assess results robustness, the individualized-conductivity models were also examined for their sensitivity to the cluster-forming threshold: for both the amplitude and relative topography analyses, second-level results were additionally thresholded at four further cluster-defining thresholds (uncorrected *p* < 0.01, 0.005, 0.0005, and 0.0001), alongside the *p* < 0.001 threshold used as the reference. At each threshold, peak-location shift was computed as the distance between the peak of that threshold’s largest surviving cluster and the peak of the largest cluster at the reference threshold for the same contrast. This approach makes the comparison robust to cluster count or ranking changes across thresholds. Suprathreshold cluster extent at each threshold is additionally reported as a fraction of the 142,799-voxel analysis mask, to give a scale-free measure of map extent alongside the raw voxel count.

## 3. Results

### 3.1. Grand Average ERP Curves

First, the grand average ERP waveforms, time-locked to both the stimulus onset and feedback presentation, were computed to ensure proper interpretation of the ERP components present in the 300–500 ms time window at Fz, Cz, and Pz. To identify systematic shifts in neurophysiological responses from childhood through emerging adulthood, additional waveforms were provided for four discrete age bins: 7–10.5, 10.5–14, 14–17.5, and 17.5–21 years.

**Figure 3:**
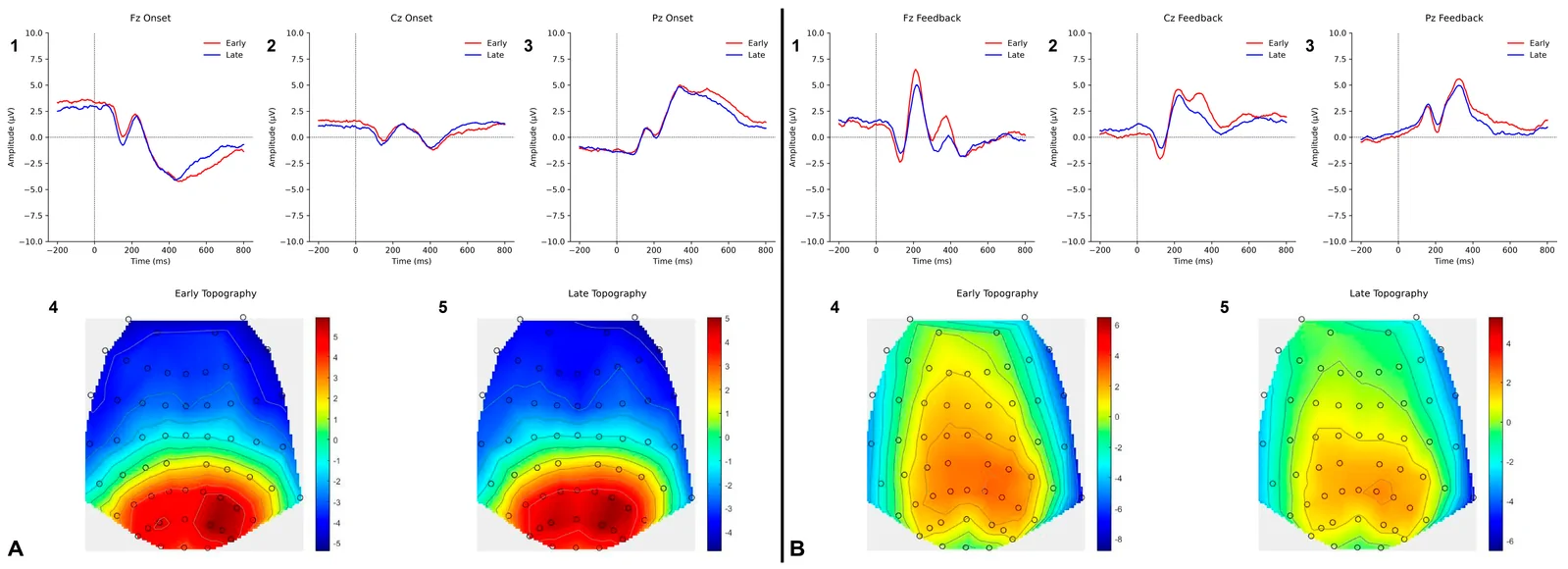
Grand average ERP waveforms across the entire sample. Results are organized into two panels representing stimulus onset-related ERP data (Panel (**A**)) and feedback presentation-related ERP data (Panel (**B**)). Within each panel, numbered elements depict: 1 (Fz channel waveform), 2 (Cz channel waveform), 3 (Pz channel waveform), 4 (Early-stage topographic map), and 5 (Late-stage topographic map). Red and blue lines in elements 1–3 represent Early and Late task stages, respectively. Topographic maps (4, 5) are oriented with anterior channels at the top and posterior channels at the bottom and display average amplitude within the 300–500 ms time window. Circles on the topographic maps (elements 4–5) mark electrode positions.

The stimulus onset-related ERP waveforms differ greatly between the anterior, central, and posterior leads. Specifically, anterior ERP features a prominent negative component in the window of interest, while in posterior ERP, a high-amplitude positive component is present, and central ERP features effectively none of them. Within each site, Early and Late events are characterized by different component amplitudes; however, the general shape of the waveform does not differ (Figure 3).

Regarding the feedback-locked ERPs, at the anterior leads, a low-amplitude positive wave is present in the 300–500 ms window, more prominent in the Early stage and blunted on the Late stage. At the posterior site, the positive component is greater on the amplitude, with Early and Late conditions featuring comparable component amplitudes. At the central site, the ERP amplitude pattern is an intermediate one (Figure 3).

**Figure 4:**
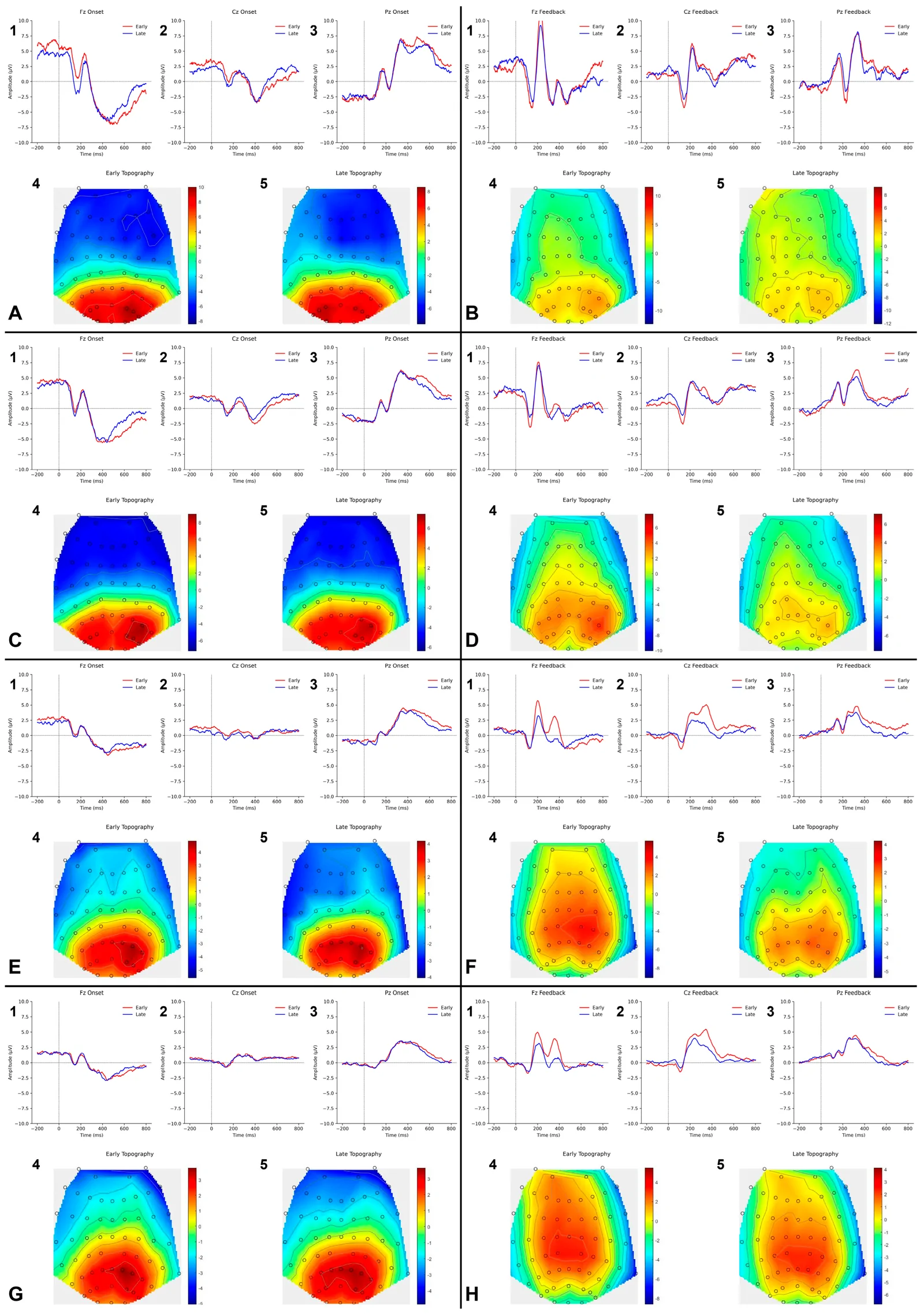
The ERP grand average waveforms within age bins. The data are categorized into four discrete age bins organized vertically: top row (7–10.5 years), second row (10.5–14 years), third row (14–17.5 years), and bottom row (17.5–21 years). Stimulus onset-related ERP results are presented in Panels (**A**,**C**,**E**,**G**) (**left** panels), while feedback presentation-related ERP results are in Panels (**B**,**D**,**F**,**H**) (**right** panels). Within each panel, numbered elements depict specific data types: 1 (Fz channel waveform), 2 (Cz channel waveform), 3 (Pz channel waveform), 4 (Early-stage topographic map), and 5 (Late-stage topographic map). Red and blue lines in elements 1–3 represent Early and Late task stages, respectively. Topographic maps (4, 5) are oriented with anterior channels at the top and posterior channels at the bottom and display average amplitude within the 300–500 ms time window. Circles on the topographic maps mark electrode positions.

Over the course of development, the amplitude of stimulus onset-related ERP activity in the 300–500 ms window was the largest in the youngest age bin. With age, both the negative wave at anterior leads and the positive wave at posterior leads substantially diminished in amplitude. The feedback presentation-related posterior positive wave within the 300–500 ms window also decreased in amplitude with participant’s age. Feedback-related positive activity present at frontal and central sites, and specifically for the Early stage, in contrast, increased with age. For all mentioned developmental changes, bins 1 and 2 and bins 3 and 4 presented with roughly similar waveforms, while bin 2 and bin 3 differed greatly in terms of the grand average waveform, regardless of event type, task stage, and examined site (Figure 4).

### 3.2. EBB Validation: Resolution-Matrix Comparison and Correlated-Source Sensitivity

Consistent with the resolution-matrix and free-energy comparisons, the following results on EBB’s suitability for this dataset were revealed. sLORETA and eLORETA reproduced their defining zero-bias property exactly (0 mm peak error, 100% of sources exact), validating the implementation (Table 1). Among the four algorithms used in the main analysis, EBB had the lowest mean peak localization error (24 mm), followed by LOR and IID (26 mm both), while GS had the highest (54 mm). EBB’s, LOR’s, and IID’s center-of-gravity errors (COG, 28–29 mm) and spatial dispersion (41–42 mm) were comparable to each other and substantially smaller than GS’s (41 mm and 65 mm, respectively). Relative to the unconstrained reference estimators, EBB’s peak localization error was lower than MNE’s (26 mm), depth-weighted MNE’s (24 mm), and dSPM’s (28 mm), though higher than the reference LCMV beamformer’s (16 mm).

EBB’s free-energy advantage over IID was not consistent across subjects. In a separate seven-subject comparison, EBB exceeded IID’s free energy in five of seven subjects (by 81 to 685 log-evidence units) but fell below it in two of seven: by 55 units in one subject and by 408 units in another, where EBB’s variance explained also dropped to 94.7% against IID’s 97.7% (subject 109). The correlated-source beamformer variant, EBBcorr, produced a meaningfully different operator from EBB (21–30% relative difference across subjects). However, the free energy of two EBB algorithms was within 3.9 to 19.8 log-evidence units of EBB’s (median ≈ 10) in six of seven subjects, indicating no consistent advantage for either variant.

### 3.3. The Consensus of Four Inversion Algorithms

**Figure 5:**
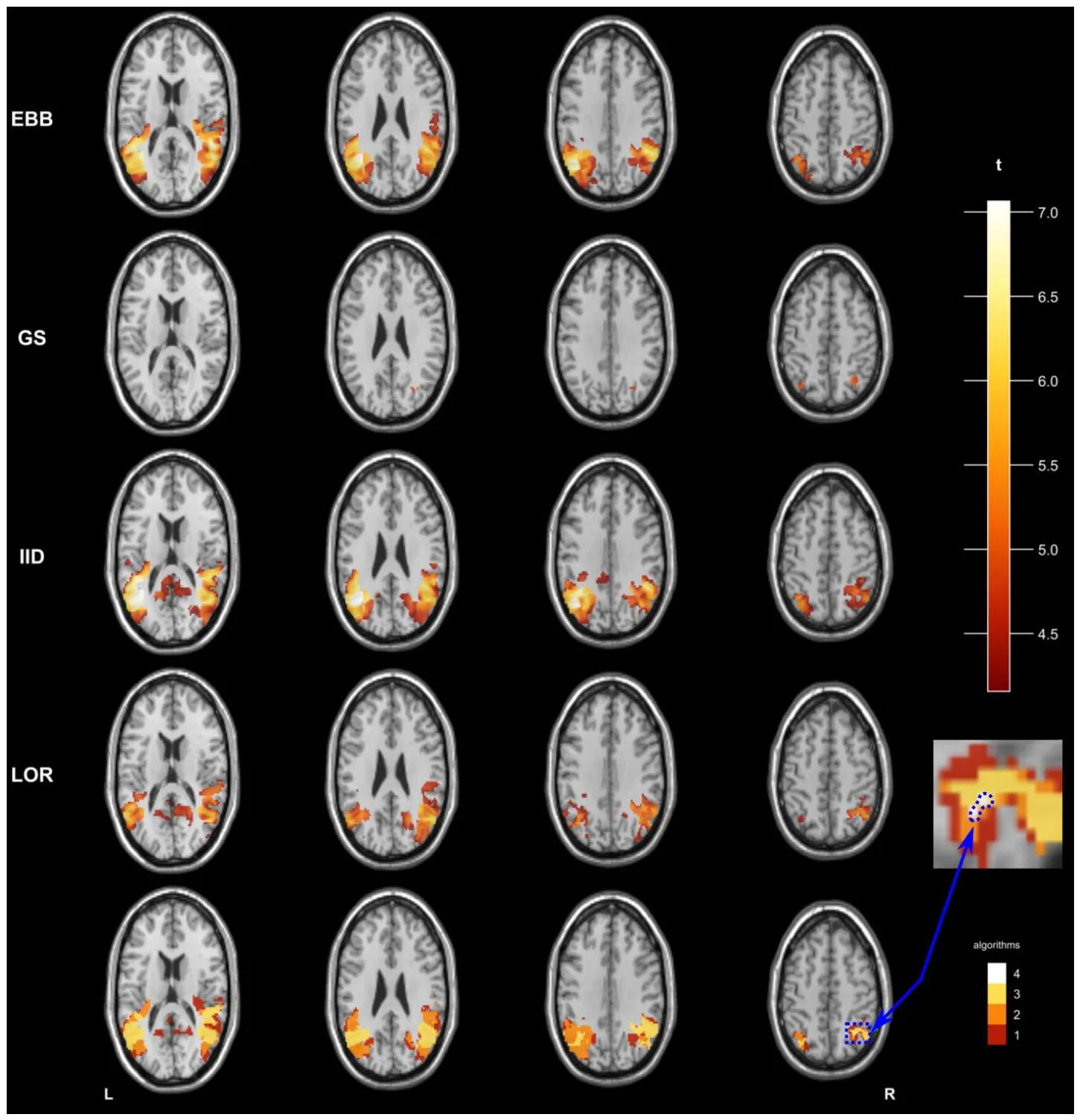
The inter-algorithm consensus map for Onset-locked events. Per-algorithm statistical montage (**top**) and algorithm-agreement map (**bottom**) for the Onset Early > Late contrast. Each algorithm (GS, LOR, IID, EBB) map is thresholded at its own whole-brain voxel-level FWE threshold (*p* < 0.05) on a shared red–white t scale. The agreement map shows the number of algorithms suprathreshold at the common voxel-level FWE threshold, with the top band corresponding to the clusters tabulated in Table 2. Blue arrows and dotted outlines mark voxels exhibiting the four-algorithm agreement. Because this cluster spans only 11 voxels, a magnified inset of the boxed region is shown alongside the rightmost panel.

For the Onset events, a single focal cluster of 11 voxels was identified, located in the right inferior parietal lobule extending into the right AG (Table 2, Figure 5); robustness of this cluster across the skull-conductivity sweep is assessed separately below (Conductivity influence). No significant clusters were found for Late > Early contrast or for relative topographic localization in the Onset contrast. The analysis also revealed no clusters related to subject’s age (positively or negatively).

**Figure 6:**
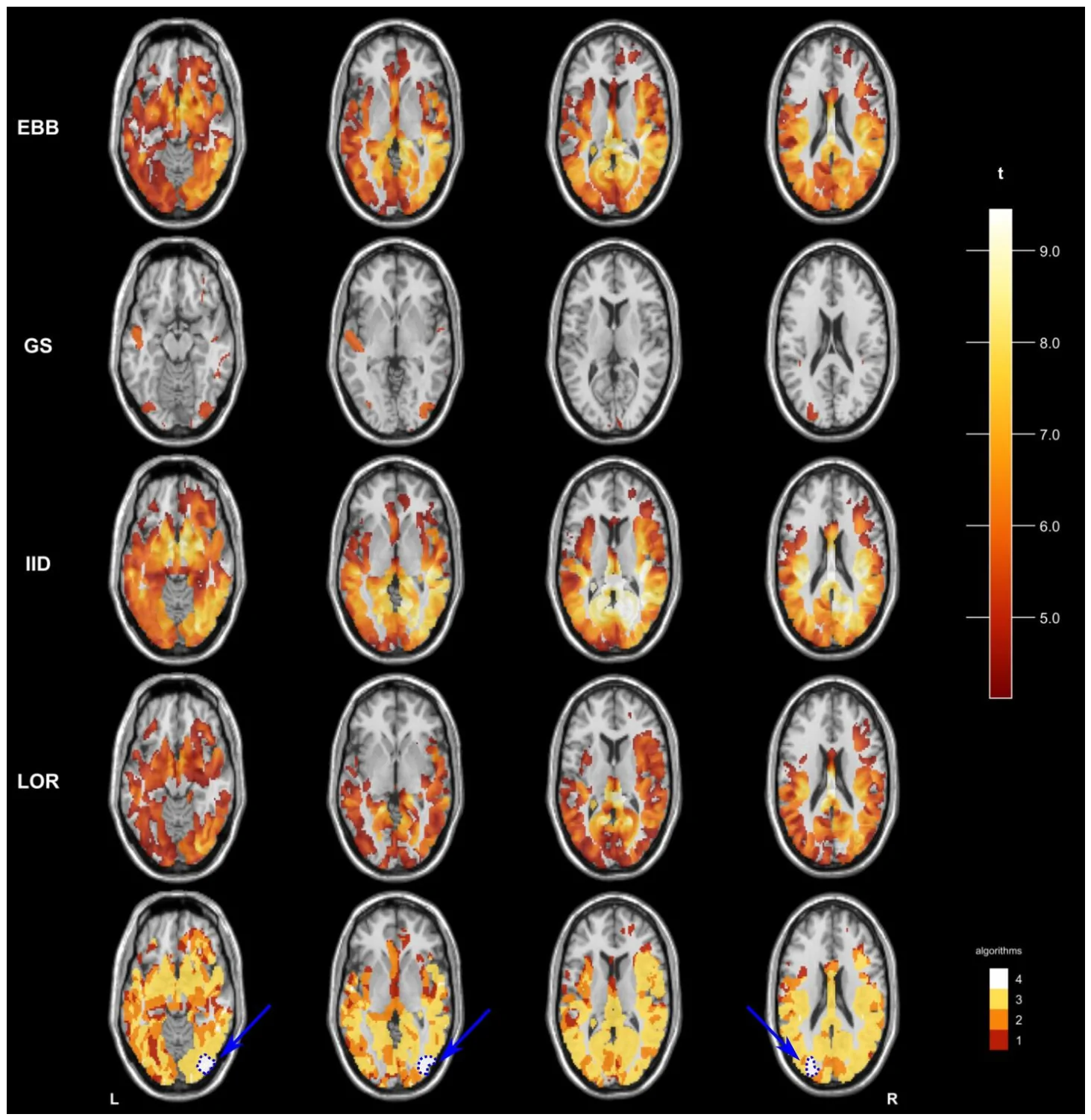
The inter-algorithm consensus map for Feedback-locked events. Per-algorithm statistical montage (**top**) and algorithm-agreement map (**bottom**) for the Feedback Early > Late contrast. Each algorithm (GS, LOR, IID, EBB) map is thresholded at its own whole-brain voxel-level FWE threshold (*p* < 0.05) on a shared red–white t scale. The agreement map shows the number of algorithms suprathreshold at the voxel-level FWE threshold, with the top band corresponding to the clusters tabulated in Table 3. Blue arrows and dotted outlines mark clusters of four-algorithm agreement.

The Feedback amplitude analysis of the Early > Late contrast demonstrated 10 clusters surviving the four-way conjunction (1389 voxels total; Table 3, Figure 6). The largest cluster comprised partial volumes of the right inferior occipital gyrus, extending into the right middle occipital, lingual, and fusiform gyri. Additional consensus clusters were localized to the left superior, middle, and inferior occipital gyri, left superior and middle temporal gyri and Heschl’s gyrus, left orbitofrontal cortex, right middle and inferior temporal gyri, and right temporal pole. Robustness of these clusters across the skull-conductivity sweep is assessed separately below (Conductivity influence). No significant clusters were found for Late > Early contrast or for relative topographic localization in the Feedback contrast. The analysis also revealed no clusters related to subject’s age (positively or negatively). In the four-way relative topographic maps conjunction analysis, no clusters survived the common voxel-level FWE threshold at the individualized skull conductivity model.

### 3.4. The Consensus of Three Inversion Algorithms: Exploratory Analysis

The Early > Late stimulus onset-related contrast consensus encompassed seven clusters, totaling 6337 voxels, dominated by bilateral AG and MTG. The Early > Late feedback-related contrast mask produced the large effect, producing a cluster of 66,462 voxels (46.3% of the analysis mask) with prominent local maxima extending across occipital, temporal, and fusiform cortices (Table 4). No consensus clusters were produced by age effects (positive or negative).

The relative topography analysis based on scaled images provided additional clusters of three-algorithm consensus. The Early > Late contrast for onset-locked events yielded four clusters (684 voxels total) located primarily at the left MTG and right AG. For feedback-locked events, Early > Late contrast produced four clusters (703 voxels total) with the peaks in the right middle occipital gyrus and the middle and inferior temporal gyri. Unlike in amplitude analysis, the Late > Early contrast for feedback-locked events, also retrieved eight clusters, totaling 26,372 voxels with one primary cluster of 24,486 voxels (17.1% of the mask) in right middle cingulate cortex, left paracentral lobule, and ACC. Smaller clusters were located in the bilateral insula, left precentral, supramarginal, and lingual gyri, and right AG (Table 5). Other relative topography analyses produced no findings.

### 3.5. Conductivity Influence

The four-way and dense-three consensus contrasts reported in Table 2, Table 3, Table 4 and Table 5 above survived at all six fixed conductivities tested (k ranges given in each table).

For the stimulus onset-related Early > Late four-way conjunction, spatial overlap with the individualized model’s cluster (11 voxels, Montreal neurological institute coordinates, MNI 34, −56, 50) was low at every fixed conductivity (Dice = 0.000 at 0.003–0.006 S/m; 0.042 at 0.007 S/m; 0.022 at 0.008 S/m). Matching each fixed-conductivity map’s cluster nearest to the individualized-model peak produced displacements of 10.0–43.5 mm (0.43–1.87× the map’s own FWHM; Table 6), with three of the six fixed conductivities (0.003, 0.004, 0.006 S/m) shifted by more than one FWHM. Thus, a significant four-way cluster (or multiple, up to 25) was present at every conductivity tested, but not always near the individualized-model peak.

For the Feedback paired Early-versus-Late four-way conjunction, the individualized model’s cluster (1389 voxels, peak MNI 40, −76, −2) showed Dice coefficients of 0.19–0.25 against the six fixed conductivities (Table 7). Matching each fixed-conductivity map’s cluster nearest the individualized model’s main cluster gave displacements of 8.2–19.4 mm (0.34–0.80× the map’s own FWHM)—within one FWHM at every fixed conductivity—with matched peak t of 6.32–7.63, all exceeding the individualized model’s peak t (6.07).

### 3.6. Spatial Agreement Between the Inversion Algorithms’ Results

The GS’s agreement with each of the other three algorithms was statistically indistinguishable from expected by chance (amplitude Dice ≤ 0.10, permutation *p* ≥ 0.002; topography Dice = 0.00, permutation *p* = 1.0 for all three GS pairs). LOR, IID, and EBB agreed with each other above chance in both analyses (amplitude Dice 0.46–0.54; topography Dice 0.29–0.72; permutation *p* = 0.001, the floor of the test, for every LOR/IID/EBB pair).

### 3.7. Smoothing and Cluster Forming Threshold Influence

The spatial overlap between the maps constructed with additionally smoothed data and ones based on the base (8 mm FWHM) level was: median Dice 0.98 at 8.25 mm, 0.96 at 8.94 mm, 0.89 at 12 mm, and 0.82 at 16 mm, with median peak-location shift ranging from 2.8 mm to 27.9 mm (non-monotonically) over the same range (Table 8).

In the cluster threshold influence analysis, median peak location shift relative to the *p* < 0.001 reference threshold was 0.0 mm at every threshold tested, in both analyses (Table 9).

Maximum peak-location shift across the five thresholds, by contrast, is given in Table 10; as above, this reflects the comparison of the largest clusters rather than the best-aligning ones. In the paired-samples design used throughout, the largest shifts occurred for the Early > Late contrast in both analyses (up to 116.0 mm for amplitude, 109.2 mm for relative topography) and for the relative-topography Late > Early contrast (up to 87.7 mm); the amplitude Age (negative) contrast showed a smaller maximum shift (23.4 mm). Wherever the amplitude Age (positive) or relative-topography Age (negative) contrasts produced a surviving cluster, its peak did not move across thresholds (0.0 mm); the relative-topography Age (positive) contrast never produced a surviving cluster at any threshold tested and is therefore omitted from Table 10.

### 3.8. Electrode Displacement Influence

**Figure 7:**
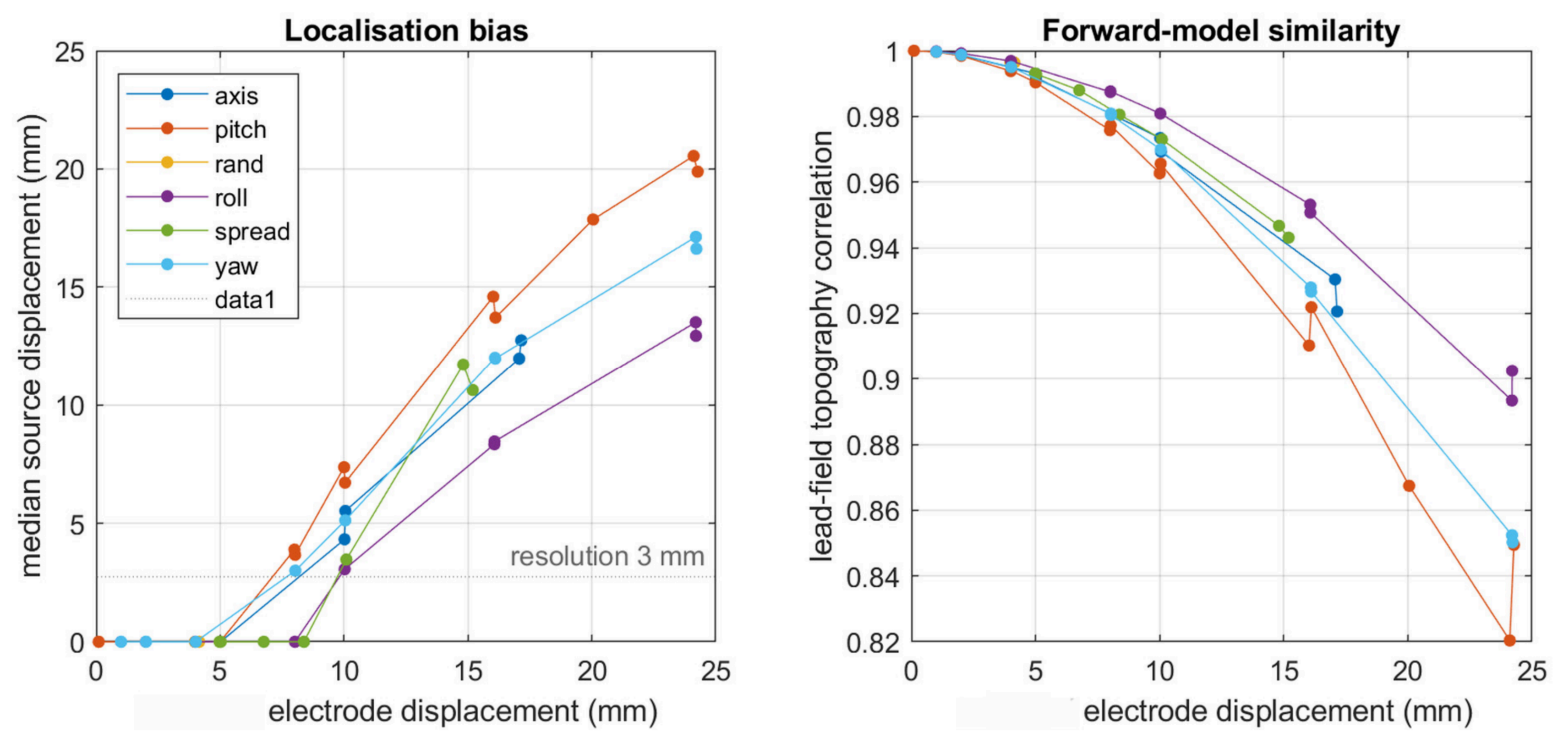
Sensitivity of EEG source localization to electrode cap displacement. (**Left**): Median source displacement (mm) as a function of electrode displacement (mm) for different rotational modes. (**Right**): Lead-field topography correlation (reliability) as a function of electrode displacement (mm).

As a result of perturbation analysis of electrode cap displacement, at 1 mm displacement, topography correlation was 0.9996, decreasing to 0.990 at 5 mm, 0.963 at 10 mm, and 0.867 at 20 mm displacement (Figure 7). Analysis of rotational perturbation modes at 10 mm displacement revealed differential sensitivity across axes. Pitch rotation (forward/backward rotation around the left–right axis) produced the greatest decrease in topography correlation (7.4), followed by yaw (5.1), spread (3.5), and roll (3.1). Prior work similarly reports that electrode position inaccuracies in the 5–10 mm range can degrade beamformer performance and source reconstruction accuracy [49]. Topography correlation at fixed 10 mm displacement showed a strong positive correlation with participant age (r = 0.737), demonstrating the same metric shift has larger influence in younger participants.

Across the nine arm-algorithm combinations tested (Table 11), voxel-level FWE-corrected *p* < 0.05 suprathreshold extent ranged from 5411 to 11,857 voxels for the Onset contrast and 64,984 to 87,380 voxels for the Feedback contrast among the eight LOR arms and was 200 (Onset) and 5869 (Feedback) voxels for the single GS arm (20 mm pitch). No arm produced zero suprathreshold voxels for either contrast, and shift magnitude did not decrease the maps extent at least for LOR inversion algorithm.

Peak location and spatial overlap (Dice coefficient of the voxel-level FWE-corrected *p* < 0.05 suprathreshold set) against the floor (0.1 mm) arm of the same algorithm are given in Table 12. For the Feedback contrast, Dice against the floor arm ranged from 0.774 to 0.949 across the seven non-floor LOR arms; peak MNI location stayed within 20 mm of the floor arm’s peak (8, −24, 30) in six of seven arms, and shifted furthest at the 20 mm pitch arm (20, −58, 14; Dice = 0.774). For the Onset contrast, Dice against the floor arm ranged from 0.367 to 0.904; peak MNI location moved from (46, −64, 16) at the floor arm to (−42, −44, 10) at the 20 mm pitch arm, a change in hemisphere, with Dice = 0.367 at that arm.

## 4. Discussion

This study utilized the ERP data combined with individual structural MRI to spatially map the associative learning-related activity in the P300 time window putatively marked with the largest cognitive effort. Specifically, we tested whether associative-learning regions such as the MTG and AG show greater P300 amplitude localization during the early, rule-acquisition stage than the late, rehearsal stage (Hypothesis 1), and whether the magnitude of this early-versus-late difference in the MTG and AG is negatively correlated with participant age (Hypothesis 2). By jointly analyzing the solutions of four distinct source reconstruction algorithms, we sought to distinguish neural mechanisms of rule acquisition from artifacts of certain mathematical inversion approaches. Given one of the four, built on a sparse spatial prior, returns a far more restricted and differently located pattern of activity than the other three, we treat agreement across all four algorithms as the more conservative and geometrically narrower evidentiary standard, and agreement across the three distributed-prior algorithms as a complementary, less conservative view of the same contrasts.

Provided the relatively young sample and change in skull conductivity with age, influencing the localization accuracy, we employed an approximation of probable individual skull conductivity we used in the main model of our study. Additionally, we demonstrated acceptable changes in main results cluster across fixed 0.007–0.008 S/m skull conductivity for stimulus onset-related data and across fixed 0.003–0.008 S/m for feedback presentation-locked ERP data. We provided support for the results’ robustness under minor changes of the smoothing kernel size and within 1 cm electrode cap shift. Last, given the beamformer usage is problematic with the correlated source, we demonstrated the EBB implementation in the current study non-inferiority to specific EBB modification for correlated sources. The aforementioned checks gave support to the results’ reliability.

### 4.1. Rule Acquisition Stage vs. Rehearsal Stage Results

Prior to discussing the localization results per se, we should first consider, which ERP component our 300–500 ms window actually captures. In our data, the sensor-level positive and negative deflections within this window overlap substantially in latency and cannot be clearly resolved from one another. For feedback-locked events, no such overlap is evident, and the localized activity is therefore most confidently attributable to the P300. However, for onset-locked events, the window may capture a mixture of posterior P300 and anterior N400 influences. Therefore, one might treat the onset-based ERP activity localization results as related to a mixture of two ERP sources. Nevertheless, the temporo-parieto-occipital localization of the onset-locked clusters is more consistent with the posterior generators typically reported for the P300 than with the anterior generators typically reported for the N400. To add, previous studies localized P300 similarly [3,50,51], suggesting that the P300 is a more plausible source of the localized activity than N400.

In the contrast between the early and late learning stages, the Onset-locked and Feedback-locked results showed different patterns. For Onset events, the three-algorithm consensus pointed to a broad, left-lateralized temporal-parietal-occipital areas during early rule acquisition, encompassing both the MTG and AG. The agreement across all four algorithms narrowed this to a single small cluster in the right inferior parietal lobule and AG, no longer including the left MTG. For Feedback events, the three-algorithm consensus identified an extensive, largely occipital-dominant cluster, while the four-algorithm consensus resolved this into ten discrete clusters spanning occipital, temporal, and orbitofrontal cortex. In both cases, the relative-topography analysis broadly corroborated the amplitude-based three-algorithm clusters—left temporal-parietal foci for Onset and occipital-temporal foci for Feedback—supporting that these patterns are not simply artefacts of raw amplitude scaling. Thus, both onset- and feedback-locked P300 localization results provide strong evidence to the hypothesis 1 of the current study.

Previous P300 source localization results, particularly within associative and reward-based learning paradigms, frequently point to a distributed network [3,9,52,53]. While the earlier feedback-related negativity (FRN) or P3a components often localize to anterior midline structures like the ACC responsible for prediction error processing [6,7,50], the later P3b component is localized to temporo-parietal areas associated with memory updating and context integration [3,50,51]. fMRI studies of trial-and-error learning and rule acquisition primarily highlight the prefrontal cortex for executive control [11,26,29] and the basal ganglia (particularly the striatum) for reward and feedback processing [26,28,54]. However, some fMRI investigations have also noted the involvement of lateral temporal and parietal cortices during rule learning and outcome processing [31,33,34]. Our EEG source reconstruction primarily localized activity in the between-stage contrasts to the lateral temporal and parieto-occipital cortices rather than the striatal core of the frontostriatal loop, which is consistent with P3b localization literature [51,52]. A smaller orbitofrontal cluster in the Feedback four-algorithm consensus (Table 3) does, however, indicate some direct possible engagement of reward-related frontal cortex alongside the lateral-temporal-dominant pattern, more so than the majority of frontostriatal-focused fMRI investigations’ results would suggest [28,54].

This divergence of our findings can be attributed to several factors specific to our experimental design and modality. First, EEG source localization is inherently limited in its ability to resolve deep subcortical structures like the basal ganglia, compared to fMRI [15,16,35]. Furthermore, our pipeline utilized an explicit cortical mesh mask applied within SPM12 during the EEG source inversion process, constraining the solution space to cortical generators. Accordingly, our results partially match the preceding ERP localization results [24,25,55] that shared the cortical constraints of the solution, and not fMRI results [2] free of such bias. Second, unlike previous studies [6,27], our paradigm lacked monetary incentive, providing rewarding feedback on trial correctness only. This might have shifted the neural processing away from dopaminergic reward-prediction error networks and toward explicit semantic and spatial rule updating, which heavily relies on the MTG and parietal cortex [32,56]. While there could be striatal involvement, it is likely diminished due to the associative learning task utilized, and the methods used in the current study are constrained to detect cortical generators. Finally, our temporal window of interest (300–500 ms), encapsulating the classic P300 or N400 components, is deeply associated with context updating, semantic integration, and memory retrieval rather than early sensory processing or late motor execution [8,57]. Thus, our results align well with previous cognitive models and may provide support for the role of declarative memory networks during early explicit rule acquisition, which subsequently disengage as the task becomes automated [58,59].

### 4.2. Age Differences

For the participant age covariate, no cortical source-level activity survived the stringent four-algorithm consensus criterion in either direction, at either the onset- or feedback-locked window. Two factors may contribute to this apparent discrepancy with prior work. First, several of the developmental studies cited above report effects at the sensor or scalp level or use different tasks and samples; our own. Interestingly, sensor-level grand-average data (see below) do show the expected age-related amplitude changes, so the discrepancy is specific to the source-level, multi-algorithm consensus step rather than to the presence of a developmental effect in the raw signal, so the age effect does not translate to the inversion, multi-inversion conjunction, or statistical model level. Of particular second interest is the second-level model that includes Good trial count and SNR as covariates of no interest alongside Age. These two regressors and trial count in particular were moderately correlated with participant age (r ≈ 0.4) in this sample, and their shared variance between Age and these covariates could reduce the model’s power to attribute source-level variance specifically to Age.

Due to preceding results, younger children often rely on external feedback to guide behavior, which is indexed by stronger anterior/medial frontal activity [13], whereas adolescents and adults show stronger parietal P3b responses associated with internal rule representation and efficient context updating [12,13]. Likewise, developmental fMRI studies highlight a gradual shift from reliance on subcortical networks to mature frontoparietal cognitive control networks during feedback-guided learning [11]. The present sensor-level grand-average ERP data are broadly consistent with this developmental literature, showing clear amplitude changes with age at both the onset- and feedback-locked windows. However, we cannot identify a specific cortical generator for this developmental change with comparable confidence, contrary to the second hypothesis of our study. We therefore treat the anatomical substrate of the ERP’s maturation in this dataset as an open question for future, more targeted source-localization work.

### 4.3. Functional Significance of the Cortical Regions of Interest

A robust finding in the exploratory, three-algorithm spatial convergence analysis was left-lateralized MTG and AG engagement during the early, active rule-search stage compared to the late, rehearsal, or rule-exploitation phase (Onset Early > Late contrast); the MTG component of this pattern did not survive when the sparse-prior algorithm’s estimate was additionally required to agree, though the four-algorithm consensus for this contrast still converged on a nearby right inferior parietal lobule/AG cluster (Table 2). The MTG is implicated in semantic memory retrieval, declarative knowledge representation, and language processing, and the AG in semantic control and multimodal integration, both part of the broader semantic-control network [60,61]. In our associative learning paradigm, the early learning stage requires participants to actively generate, hold in mind, and test covert logical rules (e.g., “always left” or “contrary to prompt”). Recruitment of these regions during the 300–500 ms window in the early stage is consistent with a reliance on semantic processing and declarative memory to evaluate these rules. Once a rule is successfully acquired and participants transition into the late rehearsal stage, the cognitive demand plausibly shifts from active hypothesis testing to automated motor mapping [59], thereby reducing this engagement.

The Feedback-locked contrast shows a different pattern: the three-algorithm consensus converged on a very large, spatially non-specific posterior occipito-temporal cluster, while the four-algorithm consensus resolved this into several more discrete regions that still include bilateral MTG and left superior temporal gyrus/Heschl’s gyrus (Table 3) alongside occipital and orbitofrontal clusters. This pattern suggests a comparatively robust, low-level visual and feedback-evaluation network alongside a more consistently recovered temporal component. Unlike the stage contrast, the age covariate did not yield a comparably robust or algorithm-independent cortical hub under either evidentiary standard (see Age Differences above). We therefore consider a functional claim supported for the stage effect, but not for the age effect.

### 4.4. Divergence of Source Inversion Algorithms

While in the two analyses discussed above the map convergence provided an estimate of a source for the target cortical activity, the differences between the four mathematical models are themselves informative about the limits of a multi-algorithm approach, and about how that approach should be weighted. The differences in mathematical priors underlying these algorithms led to vastly different spatial topographies. The GS algorithm, which heavily penalizes widespread activity [20], yielded highly focal, discrete patches of activation restricted to a smaller fraction of the searchable cortical volume than the other three algorithms. This focality is a direct consequence of its restricted search space and sparse prior. Independent characterization of all four operators’ resolution properties showed the sparse-prior algorithm’s peak-localization performance to be the lowest of the four. Additionally, its spatial agreement with other three algorithms was statistically indistinguishable from what chance placement would produce. The sparse prior therefore might trade sensitivity and locational reliability for a conservative false-positive profile and not being a more precise instrument than three other inversion algorithms.

Conversely, the IID and LOR models produced massive, smooth clusters exceeding a thousand voxels. Because these models assume equal prior variance or spatial smoothness without enforcing sparsity [17,19], they captured the widespread nature of the ERP signal though suffered from low spatial resolution, making it difficult to differentiate discrete functional nodes. Finally, the EBB approach, which leverages data-driven spatial filtering [22], achieved the best average peak-localization accuracy of the four algorithms in the same independent validation. It was also the algorithm most likely to additionally flag extensive bilateral territory, including areas that the smoother distributed methods under-represented. Nevertheless, this is a property of its distinct, empirically derived spatial filter, not necessarily greater ability in detecting a true network the other methods miss. Relying on any single one of these algorithms in isolation would have drastically altered the physiological interpretation of the data, framing rule learning as a strictly focal process if GS alone were trusted, a globally diffuse one if IID or LOR alone were trusted, or a more extensive bilateral network than the other methods support if EBB alone were trusted.

### 4.5. Limitations and Future Directions

Several methodological considerations and limitations should be acknowledged when interpreting the results of this study. While our multi-algorithm source reconstruction approach significantly enhances the reliability of the cortical estimates, EEG source localization is inherently limited by its spatial resolution and depth sensitivity [15,16]. Our methodology cannot reliably capture the deep subcortical dynamics such as those in the basal ganglia that fMRI literature strongly implicates in reward processing and feedback-guided learning [28,54]. Use of multimodal neuroimaging methods, including simultaneous EEG-fMRI, may be considered for future studies to better resolve these discrepancies. Further, even with use of an anatomical MRI for improved localization, the perturbation analyses indicated that while the statistical significance of the principal clusters was generally robust to realistic coregistration error, the precise anatomical location of the smaller, more marginal onset-locked effect might lack stability under the most extreme displacement tested (though well above realistically expected). Confidence in the anatomical specificity of a given result should scale with its effect size and consensus strength. Therefore, a small cluster from the conservative four-algorithm strategy, while holding significance, should be interpreted with caution regarding its exact location.

A notable finding that may be considered a limitation is that the four inversion algorithms are not interchangeable, equally reliable estimates of one underlying source. Independent characterization of the algorithms’ resolution properties showed that the sparse-prior algorithm localized the ERP component relatively poorly and lacked spatial agreement with the other three. Therefore, four-way agreement, while it provided conservative localization estimation, might underpower the analysis. Reporting both a strict, four-algorithm result and a more permissive, three-algorithm exploratory result, as done in the current study, makes that trade-off visible. However, this strategy does not provide us with optimal criterion for a given contrast, and simulation-based or intracranial validation would be needed to adjudicate this directly. Related to the issue of the non-interchangeable algorithm results, our developmental (age) analyses did not yield a source-level result that survived the same stringent evidentiary standard applied to the learning stage contrast, despite a clear developmental change being present in the sensor-level ERP itself. We are therefore unable to offer an empirically well-supported anatomical account of that developmental trajectory in this dataset and treat it as an open question rather than a settled finding, in contrast to what a single-algorithm or less conservative analysis might have suggested.

It should also be noted that our associative learning paradigm lacked monetary incentives and included feedback on trial correctness only. The absence of an explicit reward might have influenced the representation of reinforcement circuits in our results [26,34] that could stress the impact of declarative and semantic brain regions [60,61] observed in our data. Future research could directly compare monetary versus non-monetary feedback within the same framework to disentangle these circuits. Finally, the cross-sectional nature of our sample would restrict causal inference about any intra-individual developmental trajectory. A longitudinal design utilizing a similar multi-inversion EEG pipeline would provide deeper insight into how the neural generators of the P300 functionally mature and reorganize within an individual over time.

## 5. Conclusions

This study characterizes the neural dynamics of associative learning across development using a multi-algorithm EEG source reconstruction pipeline. Rule acquisition (Early versus Late stage) was associated with a distributed temporal-parietal-occipital network: a right inferior parietal lobule/AG cluster for cue Onset-locked activity, and a more extensive network encompassing bilateral MTG, left superior temporal gyrus/Heschl’s gyrus, and occipital and orbitofrontal regions for Feedback-locked activity, both surviving the strict four-algorithm consensus criterion. Exploratory results based on the consensus of three distributed source localization algorithms additionally implicated left MTG and AG involvement for cue Onset-locked rule acquisition specifically. Age-related differences, though present in the sensor-level ERP signal, did not resolve into a reliable, consensus-supported cortical source. Separately, the four inversion algorithms did not provide interchangeable evidence: the sparse-prior algorithm’s divergence from the other three underscores the value of reporting multiple evidentiary standards explicitly rather than relying on any single algorithm. Taken together, these results point to a distributed, largely posterior temporal-parietal-occipital network—rather than a single specialized generator—as the principal substrate of feedback-guided rule acquisition in this associative learning task.

## Figures and Tables

**Table 1:** Point-spread-function resolution metrics: six reference estimators (shared lead field) and the four algorithms’ own operators from the main analysis (mean across three sampled subjects; range across subjects in parentheses for the four SPM algorithms).

Estimator	Peak Error (mm)	% Exact	COG Error (mm)	Spatial Dispersion (mm)
sLORETA (reference)	0.0	100.0	31.6	42.8
eLORETA (reference)	0.0	100.0	24.9	34.4
MNE (reference)	25.7	0.7	26.5	39.6
dwMNE, γ = 0.8 (reference)	24.1	2.8	26.6	40.1
dSPM (reference)	27.9	0.9	35.3	45.4
LCMV, unit-noise-gain (reference)	16.3	8.1	41.0	54.9
GS (main analysis)	53.6 (52.9–54.5)	0.1	40.6	65.1
LOR (main analysis)	26.2 (25.9–26.4)	0.7	27.6	40.9
IID (main analysis)	26.2 (25.9–26.4)	0.7	27.6	40.9
EBB (main analysis)	23.9 (22.7–24.6)	1.3	28.9	41.9

**Table 2:** The four-way conjunction of the results of localization of stimulus onset-related ERP amplitude.

Cluster #	Extent (Voxels)	Peak t	Peak MNI (x, y, z)	Region(s)
1	11	4.85	34, −56, 50	R Inferior Parietal Lobule, R Angular Gyrus

The consensus of GS, LOR, IID, and EBB; common voxel-level FWE threshold *p* < 0.05, # = cluster number.

**Table 3:** The four-way conjunction of the results of localization of feedback presentation-related ERP amplitude.

Cluster #	Extent (Voxels)	Peak t	Peak MNI (x, y, z)	Region(s)
1	506	6.07	40, −76, −2	R Inferior Occipital Gyrus, R Middle Occipital Gyrus, R Lingual Gyrus, R Fusiform Gyrus
2	238	5.42	−24, −84, 34	L Superior Occipital Gyrus, L Middle Occipital Gyrus
3	191	5.81	−46, −22, 2	L Superior Temporal Gyrus, L Heschl’s Gyrus
4	168	5.25	−48, −78, −2	L Inferior Occipital Gyrus, L Middle Occipital Gyrus
5	138	5.43	−54, −40, −4	L Middle Temporal Gyrus
6	63	5.28	56, −34, 0	R Middle Temporal Gyrus
7	33	4.88	−38, 26, −18	L Anterior Orbitofrontal Cortex, L Posterior Orbitofrontal Cortex
8	31	4.79	54, −14, −20	R Inferior Temporal Gyrus
9	11	4.41	44, 18, −20	R Superior Temporal Pole, R Middle Temporal Pole
10	10	4.47	56, −46, −16	R Inferior Temporal Gyrus

The consensus of GS, LOR, IID and EBB; common voxel-level FWE threshold *p* < 0.05, # = cluster number.

**Table 4:** The three-way conjunction of the results of localization of stimulus onset- and feedback presentation-related ERP amplitudes.

Contrast	Cluster #	Extent (Voxels)	Peak t	Peak MNI (x, y, z)	Region(s)
Onset, Early > Late	1	2232	6.32	−50, −62, 20	L Angular Gyrus, L Middle Temporal Gyrus, L Middle Occipital Gyrus
	2	1004	5.31	56, −36, 18	R Middle Temporal Gyrus, R Superior Temporal Gyrus, R Angular Gyrus, R Inferior Parietal Lobule
	3	34	4.39	44, −80, 18	R Middle Occipital Gyrus;
	4	25	4.42	52, 18, 4	R Inferior Frontal Gyrus, Triangular Part, R Inferior Frontal Gyrus, Opercular Part
	5	19	4.45	40, −82, −2	R Inferior Occipital Gyrus, R Lingual Gyrus
	6	12	4.34	52, 8, −2	R Rolandic Operculum, R Insula
	7	9	4.28	−42, 26, −16	L Anterior Orbitofrontal Cortex, L Posterior Orbitofrontal Cortex
Feedback, Early > Late	1	66,462	9.62	44, −76, −2	R Inferior Occipital Gyrus, R Lingual Gyrus, R Fusiform Gyrus, R Middle Occipital Gyrus, R Calcarine Fissure

The consensus of LOR, IID and EBB; common voxel-level FWE threshold *p* < 0.05, # = cluster number.

**Table 5:** The three-way conjunction of the results of localization of stimulus onset- and feedback presentation-related ERP relative topographies.

Contrast	Cluster #	Extent (Voxels)	Peak t	Peak MNI (x, y, z)	Region(s)
Onset, Early > Late	1	412	4.97	−50, −62, 20	L Middle Temporal Gyrus, L Angular Gyrus
	2	250	4.84	48, −62, 24	R Angular Gyrus, R Middle Temporal Gyrus, R Inferior Parietal Lobule, R Middle Occipital Gyrus
	3	12	4.34	60, −30, 14	R Superior Temporal Gyrus;
	4	10	4.38	−40, −42, 14	L Superior Temporal Gyrus;
Feedback, Early > Late	1	492	5.14	46, −82, 2	R Middle Occipital Gyrus, R Inferior Occipital Gyrus
	2	106	5.06	66, −36, −8	R Middle Temporal Gyrus;
	3	75	4.66	60, −46, −22	R Inferior Temporal Gyrus;
	4	30	4.82	−60, −30, −22	L Inferior Temporal Gyrus;
Feedback, Late > Early	1	24,486	9.35	12, −6, 42	R Middle Cingulate Cortex, R Precuneus, L Precuneus, L Middle Cingulate Cortex
	2	1142	7.63	−30, −22, 12	L Insula, L Rolandic Operculum, L Heschl’s Gyrus, L Putamen
	3	363	6.74	36, −12, 14	R Insula, R Putamen
	4	165	6.66	−32, 0, 30	L Precentral Gyrus, L Inferior Frontal Gyrus, Opercular Part
	5	70	4.94	26, −52, 36	R Angular Gyrus, R Inferior Parietal Lobule
	6	60	5.43	−12, −44, −4	L Lingual Gyrus, L Cerebellum Lobule 4–5
	7	50	4.68	−30, −58, 2	L Lingual Gyrus, L Precuneus
	8	28	4.76	−50, −24, 24	L Supramarginal Gyrus, L Rolandic Operculum, L Postcentral Gyrus

# = cluster number.

**Table 6:** Onset, Early > Late (amplitude), four-algorithm conjunction:per-conductivity peak location (distance from the individualized-model peak in brackets) and peak t of the cluster matched to the individualized model’s cluster by voxel overlap, or by nearest peak otherwise, and whole-map spatial overlap (Dice) against the individualized-model result. GS, LOR, IID, and EBB; each conductivity thresholded at its own common voxel-level FWE threshold (*p* < 0.05). * indicates clusters considered not to correspond to the one from the individual skull conductivity model.

Conductivity	k (Voxels)	Peak MNI (x, y, z)	Peak t	Dice vs. Individual
**Individual conductivity**	11	34, −56, 50	4.85	reference
C 0.008	898	26, −56, 44 (Δ10.0 mm)	5.00 (Δ0.15)	0.022
C 0.007	133	24, −56, 44 (Δ11.7 mm)	5.01 (Δ0.16)	0.042
C 0.006	7	42, −82, 16 (Δ43.5 mm) *	4.81 (Δ0.04)	0.000
C 0.005	33	44, −64, 24 (Δ29.0 mm) *	4.69 (Δ0.16)	0.000
C 0.004	594	40, −62, 20 (Δ31.2 mm) *	5.22 (Δ0.37)	0.000
C 0.003	68	38, −68, 22 (Δ30.7 mm) *	4.79 (Δ0.06)	0.000

Bold indicates the individualized-conductivity reference row.

**Table 7:** Feedback, Early > Late (amplitude), four-algorithm conjunction:per-conductivity peak location (distance from the individualized-model main cluster in brackets) and peak t of the cluster matched to the individualized model’s main cluster by voxel overlap, or by nearest peak otherwise, and whole-map Dice and overlap coefficient against the individualized-model result. GS, LOR, IID, and EBB; each conductivity thresholded at its own common voxel-level FWE threshold (*p* < 0.05).

Conductivity	k (Voxels)	Peak MNI (x, y, z)	Peak t	Dice
**Individual conductivity**	1389	40, −76, −2	6.07	reference
C 0.008	6716	34, −90, −14 (Δ19.4 mm)	6.32 (Δ0.25)	0.227
C 0.007	7181	34, −82, 2 (Δ9.4 mm)	6.43 (Δ0.36)	0.245
C 0.006	5599	36, −82, 2 (Δ8.2 mm)	6.84 (Δ0.77)	0.254
C 0.005	12,105	36, −88, 4 (Δ14.0 mm)	7.63 (Δ1.54)	0.189
C 0.004	10,223	36, −90, 2 (Δ15.1 mm)	7.42 (Δ1.35)	0.204
C 0.003	5515	34, −88, 2 (Δ14.0 mm)	7.51 (Δ1.44)	0.247

Bold indicates the individualized-conductivity reference row.

**Table 8:** Smoothing-kernel sensitivity (amplitude, individualized conductivity): full dose–response from the 8 mm export kernel to 16 mm effective FWHM.

Effective FWHM (mm)	Contrasts Surviving (of 32)	Median Fraction of Mask Suprathreshold	Median Peak Shift vs. Base (mm)	Median Dice vs. Base
8 (base, export kernel)	10	0.153	0.0	1.000
8.25	9	0.163	2.8	0.979
8.94	9	0.169	4.5	0.958
12	9	0.193	27.9	0.894
16	10	0.207	11.8	0.816

**Table 9:** Cluster-forming threshold sensitivity by threshold (individualized conductivity, paired-samples design, the design used throughout the second-level analysis). ‘Contrasts surviving’ is out of the 32 algorithm × event × contrast combinations tested (4 algorithms × 2 events × 4 contrasts); see Statistical Analysis for the definitions of fraction of mask suprathreshold and of peak-location shift.

Analysis	Cluster-Forming Threshold (Uncorrected *p*)	Contrasts Surviving (of 32)	Median Cluster Extent (Voxels)	Median Fraction of Mask Suprathreshold	Median Peak Shift (mm)
Amplitude	0.01	10	37,187	0.273	0.0
Amplitude	0.005	15	8848	0.042	0.0
Amplitude	0.001 (ref.)	14	12,008	0.038	0.0
Amplitude	0.0005	13	16,444	0.044	0.0
Amplitude	0.0001	13	12,013	0.032	0.0
Topography	0.01	18	11,975	0.049	0.0
Topography	0.005	21	8771	0.034	0.0
Topography	0.001 (ref.)	17	9261	0.028	0.0
Topography	0.0005	16	8481	0.021	0.0
Topography	0.0001	15	8018	0.020	0.0

**Table 10:** Cluster-forming threshold sensitivity by contrast: maximum peak-location shift across the five-threshold sweep (individualized conductivity, paired-samples design).

Analysis	Contrast	Median Shift (mm)	90th Percentile Shift (mm)	Max Shift (mm)
Amplitude	Age, negative	0.0	0.0	23.4
Amplitude	Age, positive	0.0	0.0	0.0
Amplitude	Early > Late	0.0	94.3	116.0
Topography	Age, negative	0.0	0.0	0.0
Topography	Early > Late	0.0	12.2	109.2
Topography	Late > Early	0.0	0.0	87.7

**Table 11:** Surviving voxel counts (voxel-FWE *p* < 0.05) for Onset and Feedback contrasts across perturbation arms and algorithms.

Algorithm	Perturbation Condition	Onset Voxels	Feedback Voxels
LOR	Floor (0.1 mm)	5685	66,603
LOR	Realistic per-subject	5411	66,032
LOR	Age-graded, high	5422	64,984
LOR	Age-graded, low	6497	70,237
LOR	10 mm (pitch)	6102	70,139
LOR	13 mm (pitch)	9986	82,112
LOR	16 mm (pitch)	11,376	82,559
LOR	20 mm (pitch)	11,857	87,380
GS	20 mm (pitch), sparse prior	200	5869

**Table 12:** Perturbation arms: peak location and spatial overlap (Dice vs. the floor arm of the same algorithm) at voxel-FWE *p* < 0.05, Onset and Feedback contrasts. Peak displacement relative to the floor arm of the same algorithm is given in parentheses after each peak MNI coordinate. The GS arm has no corresponding floor (0.1 mm) arm at the same algorithm, so no within-algorithm Dice or peak-displacement value is available for it (shown as —).

Algorithm	Perturbation Condition	Contrast	Peak MNI (x, y, z)	Peak t	Dice vs. Floor Arm
**—Onset contrast—**					
LOR	0.1 mm (floor)	Onset	46, −64, 16	6.23	reference
LOR	Realistic per-subject	Onset	46, −64, 16 (Δ0.0 mm)	6.03	0.904
LOR	Age-graded, high	Onset	48, −64, 20 (Δ4.5 mm)	6.32	0.819
LOR	Age-graded, low	Onset	58, −60, 26 (Δ16.1 mm)	6.83	0.719
LOR	10 mm (pitch)	Onset	46, −64, 18 (Δ2.0 mm)	6.60	0.667
LOR	13 mm (pitch)	Onset	34, −54, 28 (Δ19.7 mm)	6.64	0.555
LOR	16 mm (pitch)	Onset	34, −56, 28 (Δ18.8 mm)	6.54	0.455
LOR	20 mm (pitch)	Onset	−42, −44, 10 (Δ90.4 mm)	6.71	0.367
GS	20 mm (pitch), sparse prior	Onset	48, −48, −14	5.69	—
**—Feedback contrast—**					
LOR	0.1 mm (floor)	Feedback	8, −24, 30	9.78	reference
LOR	Realistic per-subject	Feedback	8, −22, 30 (Δ2.0 mm)	9.83	0.949
LOR	Age-graded, high	Feedback	8, −24, 30 (Δ0.0 mm)	9.65	0.887
LOR	Age-graded, low	Feedback	8, −26, 30 (Δ2.0 mm)	9.63	0.901
LOR	10 mm (pitch)	Feedback	6, −18, 30 (Δ6.3 mm)	9.75	0.875
LOR	13 mm (pitch)	Feedback	6, −14, 30 (Δ10.2 mm)	9.45	0.816
LOR	16 mm (pitch)	Feedback	6, −14, 30 (Δ10.2 mm)	9.10	0.798
LOR	20 mm (pitch)	Feedback	20, −58, 14 (Δ39.4 mm)	9.80	0.774
GS	20 mm (pitch), sparse prior	Feedback	−52, −10, −22	6.93	—

## Data Availability

The raw data supporting the conclusions of this article will be made available by the authors on request.
